# Xanthohumol Interferes with the Activation of TGF-β Signaling in the Process Leading to Intestinal Fibrosis

**DOI:** 10.3390/nu15010099

**Published:** 2022-12-25

**Authors:** Sun-Mi Yun, Young-Min Han, Moon-Young Song, Da-Young Lee, Hyun Su Kim, Seok-Ho Kim, Eun-Hee Kim

**Affiliations:** 1 College of Pharmacy and Institute of Pharmaceutical Sciences, CHA University, Seongnam 13488, Republic of Korea; 2 College of Pharmacy, Kangwon National University, Chuncheon 24341, Republic of Korea

**Keywords:** Xanthohumol, intestinal fibrosis, TGF-β signaling, NF-κB signaling, α-SMA

## Abstract

Fibrosis has various biological processes and affects almost every organ, especially in patients with inflammatory bowel disease, including Crohn’s disease, who experience discomfort caused by intestinal fibrosis, which is a problem that needs to be resolved. TGF-β signaling is known to act as a key regulator of intestinal fibrosis, and its modulation could be an excellent candidate for fibrosis therapy. Xanthohumol (XN) has various effects, including anti-inflammation and anti-cancer; however, the detailed mechanism of TGF-β signaling has not yet been studied. The purpose of this study was to investigate the mechanism underlying the anti-fibrotic effect of XN on TGF-β1-induced intestinal fibrosis using primary human intestinal fibroblasts (HIFs). In this study, to check the anti-fibrotic effects of XN on intestinal fibrosis, we assessed the expression of fibrosis-related genes in TGF-β1-stimulated HIFs by qPCR, immunoblotting, and immunofluorescence staining. As a result, XN showed the ability to reduce the expression of fibrosis-associated genes increased by TGF-β1 treatment in HIFs and restored the cell shape altered by TGF-β1. In particular, XN repressed both NF-κB- and Smad-binding regions in the α-SMA promoter, which is important in fibrosis. In addition, XN inhibited NF-κB signaling, including phosphorylated-IkBα and cyclooxygenase-2 expression, and TNF-α-stimulated transcriptional activity of NF-κB. XN attenuated TGF-β1-induced phosphorylation of Smad2 and Smad3, and the transcriptional activity of CAGA. Particularly, XN interfered with the binding of TGF-Receptor I (TβRI) and Smad3 by binding to the kinase domain of the L45 loop of TβRI, thereby confirming that the fibrosis mechanism did not proceed further. In conclusion, XN has an inhibitory effect on TGF-β1-induced intestinal fibrosis in HIFs, significantly affecting TGF-β/Smad signaling.

## 1. Introduction

Intestinal fibrosis is a pending challenge in inflammatory bowel diseases (IBD), such as ulcerative colitis (UC) and Crohn’s disease (CD) [1]. Fibrosis of the gastrointestinal tract leads to stenosis by narrowing of the lumen, which is the final result of chronic transmural inflammation and uncontrolled wound healing, ultimately resulting in scarring and tissue distortion [2]. Fibrosis is one of the most threatening complications of CD, occurring in more than one-third of patients and causing intestinal obstruction due to repeated stricture formation [3]. Approximately 50% of patients with CD suffer from fibrotic strictures, and 75% of them eventually undergo surgery [4]. These issues, including fibrogenic complications, represent a significant portion of healthcare costs owing to the serious morbidity and mortality [5]. Cell damage, TGF-β production, recruitment of inflammatory cells, release of reactive oxygen species, activation of myofibroblasts, and collagen-producing cells are essential for the process of fibrosis [6]. Cytokines and chemokines, as well as many cellular mediators, affect the gut during fibrogenesis [7]. Inflammation plays a strong stimulating role in fibrosis, but once fibrosis is established, it is difficult to reverse it by modulating inflammation alone; therefore, regulation of key mechanisms separately from inflammation is considered to be important for fibrosis [2].

Especially, TGF-β is a cytokine involved in several organs and tissue fibrosis, including the gastrointestinal tract, and the increase in TGF-β transcripts is closely related to the phosphorylation of Smad2 and Smad3 as downstream of TGF-β signaling [8]. This signaling starts with TGF-β binding to the type III TGF-β receptor, subsequently forming a heteromeric complex with the TGF-β type II receptor (TβRII) [8]. Binding of ligands to TβRII recruits and activates the TGF-β type I receptor (TβRI) via interactions between TβRII and TβRI [8]. Activated TGF-β receptors phosphorylate Smad2 and Smad3 heterodimers, which interact with Smad4 [9]. The Smad2/3–Smad4 complex translocates into the nucleus, enhancing the transcription of pro-fibrogenic genes, such as plasminogen activator inhibitor-1 (PAI-1), fibronectin (FN), collagen type I (Col1a1), and alpha-smooth-muscle actin (α-SMA) [10]. For this reason, activation of the TGF-β mechanism has been suggested as a possible therapeutic target for intrinsic fibrosis; however, the detailed mechanism of TGF-β signaling in intestinal fibrosis is yet to be fully understood.

Xanthohumol (XN), a prenylated chalcone isolated from the inflorescences of hops (*Humulus lupulus* L.), has widespread biological functions, including anti-inflammatory, antiviral, and chemo-preventive effects against various cancers [11]. Numerous reports have suggested that natural compounds are therapeutic for the prevention of diseases, particularly inflammation and cancers [12]. In particular, studies have shown that XN reduces hepatic inflammation and the expression of pro-fibrogenic genes in a murine model [13] and inhibits TGF-β-induced cardiac fibroblast activation by regulating PTEN/AKT/mTOR signaling [11]. We previously demonstrated that XN could prevent inflammation in colitis via the downregulation of the NF-κB pathway [14]. Considering the crucial role of XN in several fibrotic diseases, we expect that the inhibition of TGF-β signaling and NF-κB by XN could prevent intestinal fibrosis. In this study, using human primary fibroblasts stimulated with TGF-β1, we demonstrated that XN can attenuate intestinal fibrosis by downregulating α-SMA transcriptional regulation via inhibition of TGF-β/Smad3 signaling.

## 2. Materials and Methods

### 2.1. Plasmids and Antibodies

We generated all constructs and all mutant constructs of TβRI and α-SMA promoter using site-directed mutagenesis and confirmed the constructs by DNA sequencing. The antibody list is described in Table 1.

### 2.2. Cell Culture and Drug Treatment

Primary human normal intestinal fibroblasts (HIFs) were isolated as previously described elsewhere [15] and kindly provided by J-H Yoo’s Lab. Cells were cultured in Dulbecco’s modified Eagle’s medium (HyClone, GE Healthcare, UT, USA) containing 10% (*v*/*v*) fetal bovine serum (ATCC, Manassas, VA, USA), 100 U/mL penicillin, and 100 μg/mL streptomycin, and were serum-starved overnight before experiments. SW620, a human colon epithelial cancer cell line, was obtained from the American Type Culture Collection (ATCC, Manassas, VA, USA) and maintained according to the ATCC’s instructions. These cells were maintained at 37 °C in a humidified atmosphere containing 5% CO_2_ and cultured in RPMI 1640 (HyClone, GE Healthcare, UT, USA) containing 10% (*v*/*v*) fetal bovine serum (ATCC, Manassas, VA, USA), 100 U/mL penicillin, and 100 μg/mL streptomycin. Transient transfections were carried out using PEI (polyethylenimine, Polysciences Inc., Warrington, PA, USA).

The synthetic method of XN was carried out as previously described [14]. The purity of XN and biotinylated XN was confirmed to be more than 95% based on NMR spectrum analysis. The biotinylated compound was a 5:1 mixture of regioisomers. XN was dissolved in dimethyl sulfoxide (DMSO, Millipore Sigma Corporation, St.Louis, MO, USA). TNF-α and TGF-β1 as inducers were purchased from Millipore Sigma Corporation (St. Louis, MO, USA). These drugs were dissolved in DDW. Cells were differentiated and treated with 5 ng/mL TGF-β1, 1 ng/mL TNF-α, and 25 μM XN, or different amounts depending on experiments.

### 2.3. Real-Time Quantitative Reverse Transcription PCR (qRT-PCR)

This assay was carried out as previously described [16]. Briefly, total RNA was isolated from cells using TRIzol reagent (Invitrogen, Carlsbad, CA, USA), according to the manufacturer’s protocol. Reverse transcription was performed with 2 μg of pure RNA using SuperScript II reverse transcriptase (Invitrogen, Carlsbad, CA, USA). Expression level-specific genes were determined by qRT-PCR (ViiATM 7 Real-time PCR system, Applied Biosystems, Waltham, MA, USA). All oligonucleotide primers, listed in Table 2, were synthesized by Macrogen (Seoul, Republic of Korea).

### 2.4. Immunoblot and Immunoprecipitation (IP) Analysis

Whole-cell lysates of mammalian cells and colon tissues were prepared and analyzed for immunoblot as previously performed [14]. For IP, cells were washed twice in cold PBS and lysed in Cell Lysis Buffer (Cell Signaling Technology, Danvers, MA, USA) plus phosphatase and protease inhibitors (Roche Applied Science, Mannheim, Germany). Whole-cell extracts were incubated with the appropriate primary antibodies overnight at 4 °C. Antibody-bound proteins were precipitated with protein A/G beads according to the manufacturer’s protocol. The beads were washed four times with lysis buffer and then eluted in 2x SDS sample loading buffer. Eluted proteins were separated by SDS-polyacrylamide gel electrophoresis (Bio-Rad Laboratories, Hercules, CA, USA), transferred to PVDF membranes (Merck Millipore), and detected using appropriate primary antibodies coupled with a horseradish peroxidase-conjugated secondary antibody using chemiluminescence (Thermo Fisher Scientific, MA, USA) and the LAS-4000 imager (GE Healthcare Life Sciences, Piscataway, NJ, USA).

### 2.5. Luciferase Assays

Human colon cancer cells in 24-well plates were transiently transfected with NF-κB or α-SMA-promoter luciferase reporter using Lipofectamine^®^ 2000 Transfection Reagent (Invitrogen, Carlsbad, CA, USA). Then, 24 h after transfection, cells were treated with 5 ng/mL TGF-β1 and 25 μM XN. Cells were collected and assayed for luciferase activity using the luciferase assay system (Promega, Madison, WI, USA) according to the manufacturer’s instructions. Each experiment was repeated in triplicate.

### 2.6. Immunofluorescence

Primary HIFs seeded on chamber slides were exposed to TGF-β1 for 24 h and then the supernatant was discarded, and the cells were incubated further for 24 h with vehicle, TGF-β1, or TGF-β1 combined with XN, respectively. Cells were fixed with 4% formaldehyde and subjected to immunofluorescence staining and then were incubated with 5% bovine serum albumin for 1 h and incubated with appropriate primary antibodies (α-SMA and vimentin) overnight at 4 °C. Antibody-bound cells were detected by Alexa Fluor 488-conjugated secondary antibody (Invitrogen, Carlsbad, CA, USA). Slides were cover-slipped and images were obtained using a Carl Zeiss LSM 880 Confocal Microscope (Carl Zeiss, Oberkochen, Germany).

### 2.7. Chromatin Immunoprecipitation (ChIP)

The ChIP experiments were performed using the SimpleChIP^®^ Enzymatic Chromatin IP Kit (Cell Signaling Technology, Danvers, MA, USA) according to the manufacturer’s instructions. SW620 cells were cross-linked with 1% formaldehyde for 15 min at room temperature, followed by addition of glycine to 0.125 M to stop the cross-linking reaction. The cell lysate was subjected to sonication (HWASHIN TECH CO., LTD, Seoul, Korea) to generate DNA fragments. Cell lysates were incubated with an anti-Smad3 antibody, anti-p65 antibody, or IgG control antibody, followed by incubation with protein G agarose beads (Cell Signaling Technology, Danvers, MA, USA). The complex was eluted by elution buffer, followed by cross-link reversion by incubating the complex at 65 °C for 2 h. DNA was purified using DNA purification columns (Cell Signaling Technology, Danvers, MA, USA). The purified DNA fragments were amplified by PCR using primers specific to p65 or Smad3 (Table 2).

### 2.8. Statistical Analysis

Results are expressed as the mean ± standard deviation (SD). Statistical analyses of the data were performed using Graphpad (GraphPad Software, San Diego, CA, USA). The statistical significance was analyzed by one-way analysis of variance (ANOVA) and statistical significance between groups was determined by Tukey’s multiple comparison test. Significance was accepted at *p* < 0.05.

## 3. Results

### 3.1. XN Inhibits Intestinal Fibrosis in Primary HIFs

**Figure 1:**
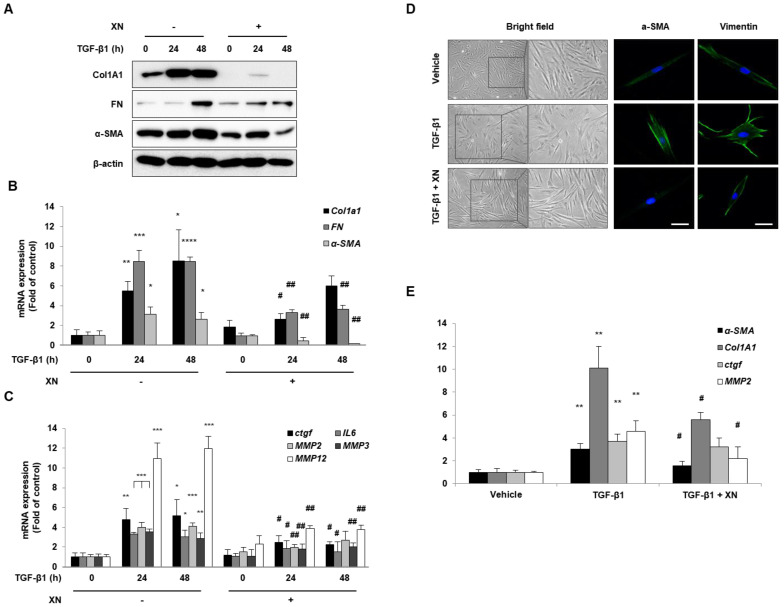
Inhibitory effects of the XN for intestinal fibrosis in primary HIFs. (**A**) Protein levels of Col1a1, FN, and α-SMA were measured by immunoblotting. (**B**) The mRNA levels of *Col1a1*, *FN,* and *α-SMA* were assayed by qRT-PCR. (**C**) The mRNA levels of *Ctgf*, *IL-6*, *MMP2*, *3*, and *12* were determined by qRT-PCR. For (B) and (C), data are means ± SD for three separate experimental samples. Data were analyzed by Tukey’s test (**** *p* < 0.0001, *** *p* < 0.001, ** *p* < 0.01, * *p* < 0.05 vs. vehicle cells; ## *p* < 0.01, # *p* < 0.05 vs. TGF- β1-treated cells). (**D**) Primary HIFs were exposed to TGF-β1 for 24 h and then the supernatant was discarded, and the cells were incubated further for 24 h with vehicle, TGF-β1, or TGF-β1 combined with XN, respectively. After the experiment, we observed cell morphology (bright field) using a microscope (magnification: ×40) and endogenous expression of α-SMA and vimentin (green), respectively. Images were examined on a confocal microscope. Scale bar: 40 μM. (**E**) The *Col1a1*, *α-SMA*, *MMP2,* and *Ctgf* mRNA levels were determined by qRT-PCR. Data are means ± SD for three separate experimental samples. Data were analyzed by Tukey’s test (** *p* < 0.01 vs. vehicle cells; # *p* < 0.05 vs. TGF-β1-treated cells).

To evaluate whether XN can prevent fibrogenesis, we identified several factors required for fibrosis progression. Primary HIFs were co-cultured with XN and stimulated with TGF-β1 for 24 or 48 h. Primary HIFs treated with TGF-β1 had increased fibrosis endpoint markers such as Col1a1, FN, and α-SMA, whereas XN-treated HIFs had decreased expression of these factors at the mRNA and protein levels (Figure 1A,B). In addition, the pro-fibrotic parameters connective tissue growth factor (CTGF) and interleukin (IL)-6 showed that the TGF-β1-induced increased expression was decreased by XN. Furthermore, we found that the expression of matrix metalloproteinases (MMP), including MMP2, 3, and 12, which regulate the imbalance between extracellular matrix (ECM) release and destruction during intrinsic fibrosis [17], increased with TGF-β1 treatment; however, they decreased with XN treatment (Figure 1C).

Intestinal fibrosis is irreversible in patients with CD; therefore, preventing or reversing intestinal fibrosis in IBD is a major therapeutic target. To determine whether XN has the reversibility of the myofibroblast phenotype, we challenged TGF-β1 for 48 h and then treated with PBS, TGF-β1, or TGF-β1 with XN as a vehicle for 24 h. After incubation, the shape of cells was observed using a microscope (magnification: ×40) and cells stained with α-SMA and vimentin were examined using a confocal microscope. According to the results, XN restored the cell shape of TGF-β1-activated fibroblasts into a spindle shape and decreased the expression of α-SMA and vimentin enhanced by TGF-β1 (Figure 1D). Moreover, XN decreased the expression of TGF-β1-induced *Col1a1*, *α-SMA*, *MMP2*, and *Ctgf* at the mRNA level (Figure 1E). These results showed that XN suppressed the TGF-β1-stimulated increased expression of fibrosis-related genes and demonstrated that both simple gene expression and fibroblast cell shape could be revitalized in primary HIFs.

### 3.2. XN Reduces Fibrotic Responses via Regulation of α-SMA Promoter

**Figure 2:**
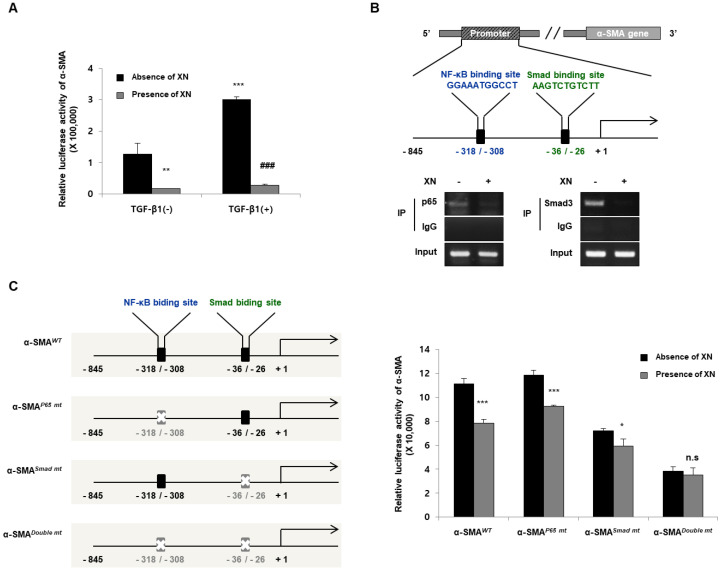
α-SMA transcriptional regulatory action of XN by reducing the expression of p65 and Smad3 in SW620 cells. (**A**) Reporter assay for α-SMA transcriptional activity. Data are means ± SD for three separate experimental samples. Data were analyzed by Tukey’s test (*** *p* < 0.001, ** *p* < 0.01 vs. vehicle cells; ### *p* < 0.001 vs. TGF-β1 treated cells). (**B**) ChIP assay using anti-p65, anti-Smad3, anti-Histone 3 (as a positive control), or anti-IgG antibody (as a negative control) and followed by PCR amplification using specific primers. (**C**) Illustration of luciferase reporters including NF-κB binding site and Smad binding site candidate regions in the α-SMA promoter sequence (**left**). Luciferase activity of α-SMA (**right**). Data are means ± SD for three separate experimental samples. Data were analyzed by Tukey’s test (*** *p* < 0.001, * *p* < 0.05 vs. absence of XN in all promoter construct-transfected cells).

α-SMA is the hallmark of mature myofibroblasts and activated fibroblasts in progressive fibrotic remodeling [18]. As shown in Figure 1, XN reduced α-SMA expression at both the protein and mRNA levels in TGF-β1-induced primary HIFs. To evaluate the transcriptional regulation of α-SMA, we challenged SW620 cells with TGF-β1 and XN. The cells were transfected with α-SMA-Luc plasmids for 24 h and treated with or without TGF-β1 and XN. We then performed a reporter assay to determine the transcriptional activity of α-SMA. XN reduced α-SMA luciferase activity independent of TGF-β1 treatment (Figure 2A). Next, to determine whether XN has an inhibitory effect on α-SMA transcriptional activity by regulating the NF-κB-binding motif at −318/−308 or the Smad-binding motif at −36/−26 contained in the α-SMA promoter, we conducted a ChIP assay. SW620 cells were subjected to ChIP using anti-p65, anti-Smad3, anti-histone 3 (as a positive control), or anti-IgG antibody (as a negative control), followed by PCR amplification using specific primers in cells treated with or without XN for 24 h. When XN was present, it blocked p65 and Smad3 binding to their motifs (Figure 2B). To verify whether the de novo NF-κB or Smad motif is necessary for α-SMA activation, we conducted site-directed mutagenesis of the α-SMA promoter, as shown in Figure 2C: α-SMA*^WT^* for vehicle control, α-SMA*^P65 mt^* for single mutation of the NF-κB-binding site, α-SMA*^Smad mt^* for single mutation of the Smad-binding site, and α-SMA*^Double mt^* for double mutation of NF-κB- and Smad-binding sites (Figure 2C). SW620 cells were transfected with various promoter constructs and treated with or without XN for 24 h. After incubation, the cells were assayed for luciferase activity to determine the α-SMA transcriptional activity. Mutation of the Smad motif also partially decreased the promoter activity of α-SMA; however, the two mutated motifs resulted in a complete reduction of α-SMA promoter activity compared to the vehicle control (Figure 2C). These results suggested that XN significantly downregulated the transcriptional activity of α-SMA by blocking both NF-κB and Smad from binding to their motifs.

### 3.3. XN Interrupts Canonical Activation of Both NF-κB and TGF-β Mechanisms

**Figure 3:**
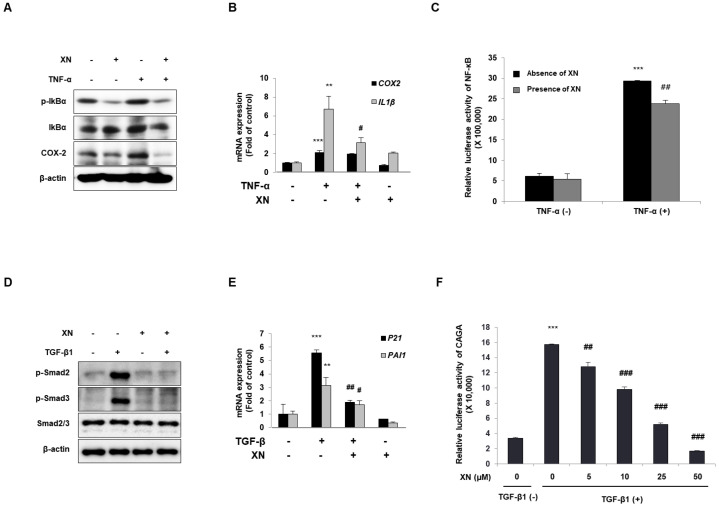
Reduction of NF-κB and TGF-β signaling pathways by XN in SW620 cells. (**A**) Protein expressions of p-IκBα, IκBα, COX-2, and β-actin using immunoblotting. (**B**) The mRNA expression of *IL-1β* and *Cox-2* using qRT-PCR. Data are means ± SD for three separate experimental samples. Data were analyzed by Tukey’s test (*** *p* < 0.001, ** *p* < 0.01 vs. vehicle cells; # *p* < 0.05 vs. TNF-α-treated cells). (**C**) Reporter assay for NF-κB transcriptional activity. Data are means ± SD for three separate experimental samples. Data were analyzed by Tukey’s test (*** *p* < 0.001 vs. vehicle control; ## *p* < 0.01 vs. TNF-α treated cells). (**D**) The expressions of p-Smad2, p-Smad3, Smad2/3, and β-actin were analyzed by immunoblotting. (**E**) The mRNA expressions of *Pai-1* and *p21* using qRT-PCR. Data are means ± SD for three separate experimental samples. Data were analyzed by Tukey’s test (*** *p* < 0.001, ** *p* < 0.01 vs. vehicle cells; ## *p* < 0.01, # *p* < 0.05 vs. TGF- β1-treated cells). (**F**) Reporter assay for CAGA transcriptional activity. Data are means ± SD for three separate experimental samples. Data were analyzed by Tukey’s test (*** *p* < 0.001 vs. vehicle control; ### *p* < 0.001, ## *p* < 0.01 vs. TGF-β1-treated cells).

The fact that XN blocks NF-κB signaling has already been reported in several studies [12,14]; therefore, we confirmed the inhibitory effect of XN under TNF-α stimulation on the NF-κB signaling pathway in SW620 cells. The cells were co-cultured with XN and then exposed to TNF-α for 24 h. XN suppressed the expression of phosphorylated IκBα and COX-2, which was increased by treatment with TNF-α (Figure 3A). To detect mRNA expression, we co-treated cells with XN and then challenged them with TNF-α for 4 h. Upon NF-κB signaling, the inhibitory effect of XN resulted in the downregulation of NF-κB target genes such as IL-1β and COX-2 (Figure 3B). Next, we transfected the SW620 cells with NF-κB–luciferase plasmids. The cells were treated with TNF-α alone or in combination with XN 24 h after transfection, and then a reporter assay for NF-κB transcriptional activity was conducted. Although TNF-α treatment increased NF-κB-mediated luciferase activity, XN treatment reduced the activity of NF-κB–luciferase in colon epithelial cancer cells (Figure 3C). These results indicate that XN exhibits anti-inflammatory effects via inhibition of NF-κB signaling, along with suppression of IκBα phosphorylation and NF-κB-mediated transcriptional activity.

As previously described, XN prevented the fibrotic effect of the TGF-β1 response by inhibiting fibrosis-related genes. α-SMA is a well-known target gene of TGF-β/Smad3 signaling [19]. Therefore, to determine whether the anti-fibrotic effect of XN is mediated by the regulation of TGF-β/Smad3 signaling, we examined Smad2/3 phosphorylation, regulation of Smad transcriptional activity, and expression of TGF-β target genes in SW620 cells. We challenged XN with TGF-β1 for 30 min (to detect Smad2/3) or 4 h (to detect mRNA levels). TGF-β greatly enhanced the phosphorylation of Smad2/3 (p-Smad2/3) (Figure 3D) and mRNA expression of *Pai-1* and *p21*, whereas XN attenuated this increase despite the TGF-β1 treatment (Figure 3E). To determine whether XN regulates Smad transcriptional activity, cells were transfected with CAGA-Luc plasmids (Smad-binding motif [20]) for 24 h and treated with TGF-β1 and XN in a dose-dependent manner. We then performed a reporter assay to assess the transcriptional activity of CAGA. CAGA-luciferase activity in TGF-β1-treated cells was increased, whereas luciferase activity decreased after XN treatment in a dose-dependent manner (Figure 3F). In conclusion, these results considered that XN blocks the TGF-β/Smad3 signaling pathway by suppressing Smad2/3 phosphorylation and CAGA-luciferase activity.

### 3.4. XN Interacts with TβRI L45 to Regulate TGF-β/Smad3 Signaling in SW620 Cells

XN contains electrophilic moieties; therefore, it is known to exert many biological effects by relying on covalent bonding to reactive protein thiols [21]. As shown in Figure 3, XN reduced the phosphorylation of Smad2/3, suggesting the possibility of regulation of TβR as an upstream pathway of Smad2/3 signaling [22].

**Figure 4:**
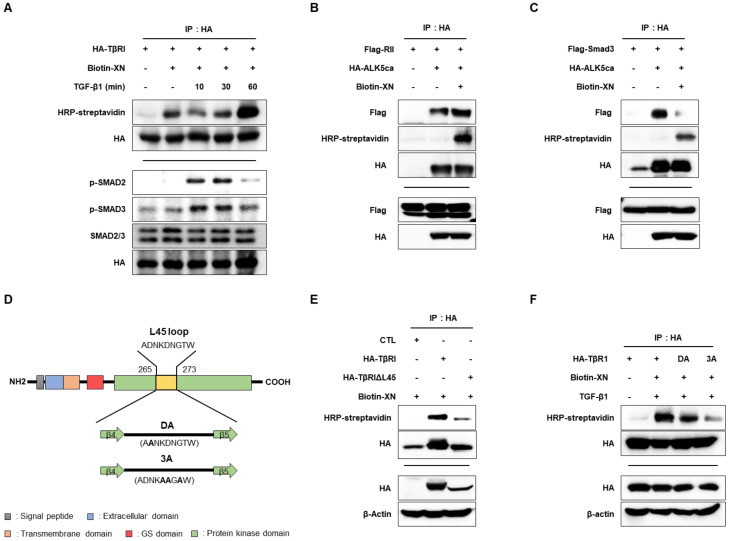
Interfering effect of interaction of TβR1 and Smad3 through binding to TβR1 by XN in SW620 cells. (**A**) HA-TβRI was transfected. IP was performed with anti-HA. IB was performed with HRP-streptavidin, HA, p-Smad2, p-Smad3, Smad2/3, and HA antibody, respectively. (**B**) Flag-RII and HA-ALK5ca were transfected. IP was performed with anti-HA. IB was performed with HRP-streptavidin, HA, and Flag, respectively. (**C**) Flag-Smad3 and HA-ALK5ca were transfected. IP was performed with anti-HA. IB was performed with HRP-streptavidin, HA, and Flag, respectively. (**D**) Protein domain structures of the L45 loop on TβR1. Domains are shown relative to their positions in the amino acid sequences. TβR1 consists of domains that include the signal peptide (gray), extracellular domain (blue), transmembrane domain (orange), GS domain (red), protein kinase domain (green), and L45 loop (yellow). DA, a single mutant (D266A) in ALK5; 3A, triple mutants (D269A, N270A, and T272A) in ALK5. (**E**) HA-TβRI and HA-TβRIΔL45 were transfected. IP was performed with anti-HA. IB was performed with HRP-streptavidin, HA, and β-actin. (**F**) Wildtype and mutants (DA and 3A) of TβRI were transfected. IP was performed with anti-HA. IB was performed with HRP-streptavidin, HA, and β-actin.

To determine whether XN interacts with TβRI, we examined its binding and Smad2/3 phosphorylation using biotin-conjugated XN. After transfecting SW620 cells with HA-TβRI, we treated the cells with biotin-conjugated XN with or without TGF-β1 for 10, 30, and 60 min. Cell lysates were subjected to IP with the HA antibody, followed by immunoblotting with HRP-streptavidin, HA, p-Smad2, p-Smad3, and Smad2/3. The binding of XN and TβRI was stronger with TGF-β1. Moreover, we showed that the phosphorylation of Smad2/3 activated by TGF-β1 was reduced 60 min after TGF-β1 treatment, with the greatest increase in the binding of XN and TβRI (Figure 4A). To determine whether XN interferes with TβRI and TβRII or binds with TβRI and Smad3, we conducted IP experiments. We transfected Flag-RII/Flag-Smad3 and HA-ALK5ca in cells, and the cells were treated with biotin-conjugated XN. Cell lysates were subjected to IP with the HA antibody, followed by immunoblotting with HRP-streptavidin, Flag, and HA. ALK5ca expression indicates TGF-β activation. XN did not interfere with the binding of TβRI to TβRII despite the presence of ALK5ca (Figure 4B), but it interfered with the binding of TβRI to Smad3 (Figure 4C). The nine-amino acid L45 sequence of TβRI was found to be essential for TGF-β signaling by docking of R-Smads [9] (Figure 4D). To further determine the TβRI motif responsible for its ability to bind XN, we generated an L45 loop deletion mutant construct of TβRI (TβRIΔL45) and examined the role of the L45 loop in the binding of XN and TβRI. HA-TβRI and HA-TβRIΔL45 were transfected in cells and the cells were treated with biotin-conjugated XN. Cell lysates were subjected to IP with the HA antibody, followed by immunoblotting with HRP-streptavidin, HA, and β-actin. The results showed that TβRIΔL45 was not associated with XN (Figure 4E). We further generated full-length TβRI constructs carrying point mutations in the L45 loop, DA (a single mutant: D266A), and 3A (triple mutants: D269A, N270A, and T272A) [20] (Figure 4D). Vehicle, DA, and 3A were transfected in cells and treated with biotin-conjugated XN and TGF-β1. Cell lysates were subjected to IP with the HA antibody, followed by immunoblotting with HRP-streptavidin, HA, and β-actin. We found that both mutants showed a reduced interaction with XN (Figure 4F). These results suggested that XN interacts with TβRI L45 to interfere with the binding of TβRI to Smad3, eventually obstructing TGF-β/Smad3 signaling.

## 4. Discussion

One of the typical complications of IBD is intestinal fibrosis, which can occur in two forms of IBD, UC and CD, but mostly in CD (>50% of patients with CD), and has a serious impact on the patient’s quality of life [23]. Intestinal fibrosis causes stenosis by narrowing the lumen, which results in scar formation and tissue distortion—requiring surgery [2]. However, intestinal fibrosis remains a difficult challenge for both basic sciences and clinicians because of the lack of medications and predictive markers for fibrosis [1,2]. Although the detailed mechanism of intestinal fibrosis is still not precisely known, there is widespread knowledge that an immune response is activated, affecting various cells in the intestine, including fibroblasts and smooth-muscle cells, to accelerate ECM accumulation and collagen deposition [24].

Inflammation is a strong stimulant that initiates fibrosis; however, once fibrosis is formed, the process is very difficult to reverse [1]. Eradication of the pathogen suppressed inflammation in a pathogen-induced IBD mouse model but did not inhibit fibrosis [25]. Our recent study validated that the pathogenesis of fibrosis is blocked when NF-κB and TGF-β signaling are inhibited together, rather than inhibiting NF-κB alone. These results suggest that it is difficult to regulate inflammation simply by regulating fibrosis. General treatment for intestinal fibrosis focuses on anti-inflammatory agents, which do not directly affect fibrosis; therefore, they may slightly regulate fibrogenesis, but cannot prevent recurrence of fibrosis [26]. Clinical data indicate that patients with IBD mainly develop late-stage stenosis, and most of them are dissatisfied after surgery; therefore, prevention of fibrosis and recurrence is important [27]. Our study confirmed that XN inhibits fibrosis-associated genes in primary HIFs, and further validated that it reverses cellular morphology even after fibrosis occurs. Therefore, these results suggest that XN may be effective in preventing the recurrence of fibrosis, as well as its anti-fibrotic role.

TGF-β isoforms, including TGF-β1, β2, and β3, are immunosuppressive cytokines that exert profound effects on the regulation of cell division, migration, proliferation, and gene expression in various cells [8]. In addition, TGF-β is known to be an important stimulator for fibroblast activation and promotes the fibrogenic phenotype, which has been proven through in vitro, in vivo, and clinical studies in various tissues such as the liver, lung, kidney, heart, and skin [8,28,29,30]. Interestingly, TGF-β isoforms are abundant in the mammalian intestine, among which TGF-β1 is the most abundant isoform [31,32]. It plays a well-known role in intestinal immunity, and TGF-β activity is involved in the development of strictures during the pathogenesis of intestinal fibrosis, leading to complications in patients with IBD, especially CD [33,34]. Intestinal stricture in patients with CD is associated with elevated TGF transcript levels and excess accumulation of ECM proteins such as collagen and FN [35,36]. Myofibroblasts isolated from the intestinal strictures of patients with CD overexpress collagen-3, and TGF-β1 promotes collagen-3 production [36]. Bruce et al. reported that TGF-β1 increased during the pathogenesis of intestinal fibrosis in the mouse intestine [37]. A recent study confirmed that inhibition of TGF-β signaling, similar to other investigations, suppresses fibrosis-associated factors. TGF-β also appears to play an important role in intestinal fibrosis.

XN is a natural product of the hop plant, and it is scientifically known for its anti-cancer, anti-inflammatory, anti-invasion, and multiple biological effects, and has been steadily studied with increasing interest [14,38,39]. In a previous study, XN was shown to have an inhibitory effect on NF-κB signaling [14,40], and recent studies on fibrosis and XN have been published [11,41,42]. According to Wang et al., XN exhibits preventive effects against liver steatosis and fibrosis caused by type 2 diabetes mellitus by regulating NRF2/AGE/RANGE/NF-κB signaling [41]. XN also reduces cardiac hypertrophy and fibrosis induced by isoprenaline via the PTEN/AKT/mTOR mechanism [11]. However, studies on intestinal fibrosis have not been clarified; therefore, we explored the role of XN in intestinal fibrosis and confirmed that XN can interrupt the development of intestinal fibrosis in vitro. According to our previous research, a covalent bond occurs between the electrophilic carbon center of the α, β-unsaturated carbonyl moiety of XN and cysteine thiol (Cys99) of IKKβ (upstream of NF-κB), leading to suppression of IKKβ/NF-κB signaling [14]. Moreover, we confirmed that XN binds to KEAP1, which has a cysteine residue-rich gene, affects NRF2 activation, and controls tumor progression. Based on the above results, in this study, we tried to check whether XN actually interacts with TGF-β-related genes when XN interfered with development of intestinal fibrosis, and we obtained evidence of XN binding to TβRI. Phosphorylation of TGF-β1 activates Smad2/3 by TβRI (also termed ALK5s), then Smad4 binds to Smad2/3 and this complex is activated, and it moves to the nucleus and transcription-related factors [10].

TGF-β signaling transduced by binding is delivered to two single-pass transmembrane receptor kinases, TβI and TβII [43]. These receptors are structurally similar and have a cysteine-rich extracellular domain, transmembrane domain, and cytoplasmic serine/threonine-rich domain [44]. When the TGF-β ligand binds to the TβII dimer, it recruits the TβI dimer and directly contacts it to form a hetero-tetrameteric complex [44,45]. TβI has a GS domain, which is composed of a series of thirty serine–glycine repeats, so serine and threonine residues within the GS domain are phosphorylated by TβII, initiating a downstream signaling cascade that activates the Smad proteins [46]. ALK5 contains a C- and N-lobe consisting of a twisted five-stranded β-sheet and a single α-helix within the N-lobe [44]. A nine-amino acid sequence between β4 and β5, the L45 loop, has an important specific sequence for Smad2/3 [44], which is suggested to serve as the docking site for Smad2/3 [47]. In addition, the L45 loop is required for the process in which TGF-β-induced changes in epithelial cells into fibroblast-shaped cells and the formation of actin stress fibers [47]. According to a study by Itoh et al., when the L45 loop of ALK5 was mutated to target the isoform that specifically binds to Smad (a single mutant: ALK5 (D266A), called ALK5 (DA), triple mutants (ALK5 (D269A, N270A, T272A), called ALK5 (3A)), phosphorylation of Smad2 and Smad-dependent reporter activity did not occur [47].

Therefore, in the present study, we validated which part of XN affected the repression of TGF signaling and, as a result, XN did not interfere with the binding of TβI and TβII but disturbed the interaction of TβI with Smad. These data inferred that XN may connect to the region near the binding site of TβI and Smad; thus, when we checked the binding site of TβI and Smad after deletion in the L45 loop, it clarified that XN did not associate anymore, which means that XN binds to the L45 portion of TβI. To determine exactly where XN binds to the L45 region, we mutated ALK5 (DA) and ALK5 (3A), known as specific sites for Smad signaling, and found that it inhibits both binding sites, especially more strongly restrained in ALK5 (3A) (Figure 4D). These results suggested that XN interrupts the binding between TβI and Smad3 by targeting the D266A, D269A, N270A, and T272A sites of ALK5. However, we did not perform a docking study. More detailed research is required in the future to accurately demonstrate where and how XN binds to block interactions and signaling cascades for intestinal fibrosis.

## 5. Conclusions

**Figure 5:**
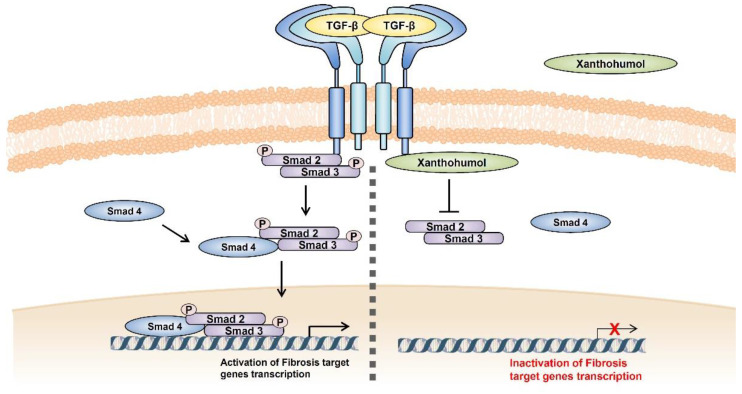
Schematic representation. Treatment of TGF-β1 aggravated the signaling of TGF-β/Smad and transcription of fibrosis-related target genes. However, exposure of XN mitigated the signaling cascade by interfering with the binding of TβRI and Smad3. “↓” Indicates induction, “┻” indicates inhibition, and “ⓟ” indicates phosphorylation.

This study is the first to reveal the role of XN in TGF-β-induced intestinal fibrosis. These results indicate that XN inhibited fibrosis-related genes and restored the cellular morphology stimulated by TGF-β1 in primary HIFs. Moreover, XN significantly blocked the NF-κB- and Smad-binding regions of α-SMA and interrupted the activation of both the NF-κB and TGF-β signaling pathways. XN interacts with the L45 loop of TβRI, thereby regulating TGF-β/Smad3 signaling. Consequently, XN alleviated the development of intestinal fibrosis induced by TGF-β1 (Figure 5). This information can be useful for development of XN as a natural functional supplement in postoperative patients. Our study suggests that XN is a novel compound beneficial for the treatment of intestinal fibrosis.

## Figures and Tables

**Table 1:** Antibodies for immunoblotting.

Name	Cat. No.	Company
α-SMA	ab7817	Abcam
β-actin	sc-47778	Santa Cruz Biotechnology
COL1A1	SP1D8	Development Studies Hybridoma Bank
COX-2	RB-9072-P1	Cayman chemical
FN	sc-8422	Santa Cruz Biotechnology
HA	sc-7392	Santa Cruz Biotechnology
Flag	F1804	Sigma
IkBα	#9242	Cell Signaling Technology
p-IkBα	#2859	Cell Signaling Technology
p65	#8242	Cell Signaling Technology
p-Smad2	#3108	Cell Signaling Technology
p-Smad3	#9520	Cell Signaling Technology
Smad2/3	#5678	Cell Signaling Technology
streptavidin	SA-5004	Vector Laboratories

α-SMA, alpha-smooth-muscle actin; β-actin, beta-actin; COL1A1, collagen, type 1 pro-peptide; COX-2, cyclooxygenase-2; FN, fibronectin; IkBα, nuclear factor of kappa light polypeptide gene enhancer in B-cells inhibitor, alpha; p65, nuclear factor-κB.

**Table 2:** Primer sequences for qRT-PCR and ChIP assays.

Species	Gene	Primer Sequence	
Human (qRT-PCR)	*18S rRNA*	Forward	GCAATTATTCCCCATGAACG
		Reverse	GGCCTCACTAAACCATCCAA
	*Col1a1*	Forward	GATTCCCTGGACCTAAAGGTGC
		Reverse	AGCCTCTCCATCTTTGCCAGCA
	*FN*	Forward	GAACTATGATGCCGACCAGAA
		Reverse	GGTTGTGCAGATTTCCTCGT
	*α-SMA*	Forward	GCAAACAGGAATACGATGAAGCC
		Reverse	AACACATAGGTAACGAGTCAGAGC
	*MMP-2*	Forward	AGCGAGTGGATGCCGCCTTTAA
		Reverse	CATTCCAGGCATCTGCGATGAG
	*MMP-3*	Forward	CACTCACAGACCTGACTCGGTT
		Reverse	AAGCAGGATCACAGTTGGCTGG
	*MMP-12*	Forward	GATGCTGTCACTACCGTGGGAA
		Reverse	CAATGCCAGATGGCAAGGTTGG
	*CTGF*	Forward	CTTGCGAAGCTGACCTGGAAGA
		Reverse	CCGTCGGTACATACTCCACAGA
	*IL-6*	Forward	AGGGCTCTTCGGCAAATGTA
		Reverse	GAAGGAATGCCCATTAACAACAA
	*IL-1β*	Forward	TTAAAGCCCGCCTGACAGA
		Reverse	GCGAATGACAGAGGGTTTCTT
	*COX-2*	Forward	TGCATTCTTTGCCCAGCACT
		Reverse	AAAGGCGCAGTTTACGCTGT
	*PAI-1*	Forward	CTCATCAGCCACTGGAAAGGCA
		Reverse	GACTCGTGAAGTCAGCCTGAAAC
	*p21*	Forward	AGGTGGACCTGGAGACTCTCAG
		Reverse	TCCTCTTGGAGAAGATCAGCCG
Human (ChIP)	*p65*	Foward	TTCTTCTTTGCATGCTACCG
		Reverse	ATGGTTTGCACATTCCACAG
	*Smad3*	Foward	CAGTGGAATGCAGTGGAAGA
		Reverse	AGGGAAGCTGAAAGCTGAAG

18S rRNA, 18S ribosomal RNA; Col1a1, collagen type 1 alpha 1 chain; FN, fibronectin; α-SMA, alpha-smooth-muscle actin; MMP, matrix metalloproteinase; CTGF, connective tissue growth factor; IL, interleukin; COX-2, cyclooxygenase-2; PAI-1, plasminogen activator inhibitor-1; p65, nuclear factor-κB.

## Data Availability

The data presented in this study are available upon request from the corresponding author.
